# Antitumor effects of pharmacological EZH2 inhibition on malignant peripheral nerve sheath tumor through the miR-30a and KPNB1 pathway

**DOI:** 10.1186/s12943-015-0325-1

**Published:** 2015-03-07

**Authors:** Pingyu Zhang, Xianbin Yang, Xiaoyan Ma, Davis R Ingram, Alexander J Lazar, Keila E Torres, Raphael E Pollock

**Affiliations:** Department of Surgical Oncology, The University of Texas MD Anderson Cancer Center, Houston, TX USA; AM Biotechnologies, 12521 Gulf Freeway, Houston, TX USA; Department of Pathology, The University of Texas MD Anderson Cancer Center, Houston, TX USA; Current address: Department of Surgical Oncology, Comprehensive Cancer Center, Ohio State University, Columbus, OH USA

**Keywords:** MPNST, miR-30a, DZNep, EZH2, KPNB1, Apoptosis

## Abstract

**Background:**

Enhancer of zeste homolog 2 (EZH2) is a key epigenetic regulator in cancer cell survival, epithelial-mesenchymal transition, and tumorigenesis. Inhibition of EZH2 has become a promising therapeutic option for various human malignancies. Previously, we demonstrated that the EZH2/miR-30d/karyopherin (importin) beta 1 (KPNB1) signaling pathway is critical for malignant peripheral nerve sheath tumor (MPNST) cell survival *in vitro* and for tumorigenesis *in vivo*. Here, we sought to determine the antitumor effects of pharmacological inhibition of EZH2 on MPNST *in vitro* and *in vivo*.

**Methods:**

We investigated the effects of an EZH2 inhibitor, 3-deazaneplanocin A (DZNep), on MPNST cell cycle, survival and apoptosis *in vitro* and on MPNST xenograft tumor growth *in vivo*.

**Results:**

We found that DZNep treatment impaired MPNST cell viability and proliferation by inducing apoptosis and cell cycle arrest *in vitro*. Consistently, DZNep treatment also reduced EZH2 and KPNB1 protein levels and upregulated miR-30d expression in MPNST cells. Intraperitoneal administration of DZNep significantly suppressed MPNST tumor initiation and growth rates in a MPNST xenograft mouse model. Immunoblot and immunohistochemical analyses showed that DZNep downregulated EZH2/KPNB1 signaling *in vivo*, thereby inhibiting MPNST tumor cell proliferation, and induced cell death. We also found that EZH2 inhibited expression of another miR-30 family member, miR-30a, in MPNST cells. Similar to miR-30d, miR-30a inhibited KPNB1 by targeting the KPNB1 3’ untranslated region in MPNST cells. Our data also showed that EZH2 suppressed miR-200b expression and induced epithelial-mesenchymal transition in MPNST cells.

**Conclusion:**

These findings demonstrated that DZNep, an inhibitor of S-adenosyl-methionine–dependent methyltransferase, suppressed EZH2/miR-30a,d/KPNB1 signaling and blocked MPNST tumor cell growth and survival *in vitro* and *in vivo*. More importantly, our study indicated that pharmacological interference of EZH2 is a potential therapeutic approach for MPNST.

**Electronic supplementary material:**

The online version of this article (doi:10.1186/s12943-015-0325-1) contains supplementary material, which is available to authorized users.

## Background

Malignant peripheral nerve sheath tumor (MPNST) is a spindle-cell malignancy that arises from peripheral nerves or deep neurofibromas [1]. MPNST behaves aggressively, has a high rate of local recurrence, and readily metastasizes to other organs. Because of its invasiveness, metastasis, and resistance to chemotherapy and radiotherapy, MPNST has a poor prognosis. Surgery and chemotherapy are the main treatments for MPNST; no targeted therapy for MPNST is available. Even with aggressive surgery and chemotherapy, MPNST patients have a 5-year survival rate of just 35%–50% [1], which indicates an urgent need for novel therapies. The use of RAS inhibitors to treat patients with plexiform neurofibromas, which give rise to NF1-related MPNSTs, has been investigated in clinical trials. In one phase 2 trial, tipifarnib, a farnesyl transferase inhibitor that blocks RAS’s ability to induce tumorigenesis, was found to be an ineffective therapy for plexiform neurofibroma, probably because RAS was activated through an alternative pathway. Therefore, novel therapeutic approaches for MPNST are urgently needed.

Enhancer of zeste homolog 2 (EZH2), a histone-lysine N-methyltransferase and a polycomb group protein, is a critical component of polycomb repressive complex 2 (PRC2), a multimeric protein complex that also includes EED, SUZ12, and RbAP46/48 [2]. PRC2 plays an essential role in the epigenetic maintenance of the repressive H3K27me3 chromatin mark [3]. Abnormal EZH2 expression has been associated with various human malignancies, including lymphoma and lung, breast, and prostate cancers [3]. In glioblastoma multiforme, EZH2 has been shown to be a functional oncogene, a prognostic factor, and a potential therapeutic target [4]. However, the role of EZH2 in MPNST pathogenesis is poorly understood. Our recent research shows that EZH2 expression is significantly higher in MPNST than in neurofibromas and normal nerve tissues. EZH2 knockdown by RNA interference in MPNST cell lines induces MPNST cell apoptosis *in vitro* and inhibits MPNST tumor growth *in vivo*, suggesting that EZH2 is a potential therapeutic target in MPNST [5].

Given the importance of EZH2 in human malignancies, multiple groups have developed pharmacological inhibitors of EZH2. One such inhibitor, 3-deazaneplanocin A (DZNep), depletes cellular EZH2 protein and is considered to be an inhibitor of S-adenosyl-methionine–dependent methyltransferase [6,7]. Studies have shown that DZNep treatment has antitumor effects in several cancer models, such as prostate cancer, leukemia, and hepatocellular carcinoma [8-10]. These data demonstrate that the pharmacological inhibition of EZH2 activity is a promising treatment for EZH2-driven cancers.

To investigate the effect of EZH2 inhibitor DZNep on MPNST cell growth and survival *in vitro*, we performed cell cycle, apoptosis, and viability analyses of MPNST cells treated with DZNep. To assess the effect of DZNep on tumor growth *in vivo*, we conducted tumor incidence and growth analyses with use of an MPNST724 xenograft mouse model. In this report, we showed that DZNep inhibited MPNST cell viability by inducing cell cycle arrest and apoptosis *in vitro*. Our results showed that DZNep also inhibited MPNST724 xenograft tumor incidence and growth rates *in vivo*. We found that DZNep depletes EZH2, subsequently induces miR-30d and miR-30a expression that inhibit Karyopherin β1 (KPNB1) in MPNST cells. In addition, we demonstrated that EZH2 transcriptionally inhibits miR-200b expression and induces epithelial-mesenchymal transition in MPNST cells.

## Materials and methods

### Cell lines and immunohistochemical and western blot analyses

Human NF1-related MPNST cell lines ST88-14, T265, MPNST642, and S462 and non-NF1-related sporadic human MPNST cell lines STS26T and MPNST724 were maintained as previously described [5]. Primary cultured normal human Schwann cells, purchased from ScienCell Research Laboratories, served as controls. Authentication of cell lines was performed by short-tandem-repeat DNA fingerprinting.

Immunohistochemical and Western blot analyses were performed according to standard protocols with minor modifications due to antibody optimization [11]. Commercially available antibodies were used for all immunoblot and immunohistochemical detection of EZH2 (1:1000, D2C9, Cell Signaling), KPNB1 (1:5000, NB100-81650, Novus Biologicals,), cleaved PARP (1:1000, ab32064, Abcam), vimentin (1:2000, RV202, Santa Cruz), E-cadherin (1:1000, H-108, Santa Cruz), Ki-67 (1:1000, MIB-1; Dako), cleaved caspase 3 (1:1000, BioCare Medical), GAPDH-HRP (1:5000, ab9483, Abcam), and actin-HRP (1:5000, I-19, Santa Cruz). Secondary antibodies were horseradish peroxidase (HRP)–conjugated rabbit anti-mouse (1:5000, Santa Cruz) and goat anti-rabbit (1:3000, Santa Cruz) antibodies for Western blot and immunohistochemical analyses.

### EZH2 inhibitor treatment, PCR analyses, cell viability assay, cell cycle analyses, and apoptosis analyses

The EZH2 inhibitor DZNep (Cayman Chemical) was dissolved in DMSO to yield the appropriate stock concentrations and then frozen at −80°C until further use. Cells were exposed to DZNep for 72 hours and then harvested for qRT-PCR as well as Western blot, cell viability, and apoptosis analyses. For qRT-PCR analyses of miRNAs, total RNA was isolated by using TRIZOL (Life Technologies) and reverse transcribed to cDNA by using the RT^2^ miRNA First Strand kit (QIAGEN). Next, q-PCR was performed with miR-200b, miR-30d, and miR-30a specific primers (QIAGEN). SNORD47 was used as a normalizing control for miRNA qRT-PCR analyses. *In vitro* cell viability was measured by using a CellTiter96 Aqueous Non-Radioactive Cell Proliferation Assay kit (Promega) according to its standard protocol. For the cell apoptosis assays, cells were stained with annexin V and propidium iodide (PI) according to the manufacturer’s protocol for an apoptosis detection kit (BD Pharmingen). For cell cycle analyses, cells were fixed and stained with PI for 30 minutes and then analyzed in a FACSCalibur flow cytometer (BD Biosciences). Data were analyzed with Cell Quest software.

### Mouse xenograft experiment

All animal procedures and care were approved by MD Anderson’s Institutional Animal Care and Use Committee. The MPNST xenograft mouse model using MPNST724 cells has been described previously [12]. For this experiment, 2 × 10^6^ MPNST724 cells were suspended in 100 μl PBS and then injected subcutaneously into the flanks of 6-week-old female hairless SCID mice. Three weeks after injection, mice were randomized into three groups (n = 9/group) to receive intraperitoneal injections of 100 μl of vehicle only, 1 mg/kg DZNep, or 5 mg/kg DZNep twice per week (Monday and Thursday) every other week. Mice were weighed, and the dimensions of their tumors were measured with calipers twice weekly. Tumor volumes were calculated by using the following equation: (length/2) × (width)^2^. Mice were monitored until their tumors were 1.5 cm in diameter or their morbidity necessitated euthanasia. Mice were killed humanely by CO_2_ inhalation, and their tumors were resected, weighed, fixed in formalin, and paraffin-embedded for H&E and immunohistochemical studies. Slides of formalin-fixed, paraffin-embedded tumor tissues from the control untreated group and the two EZH2 inhibitor–treated groups were prepared and subjected to immunohistochemical staining for cleaved caspase 3 and Ki-67. Differences in xenograft growth *in vivo* were assessed by using a two-tailed Student *t* test.

### Promoter activity analyses

A miR-30d promoter construct was generated previously [5]. Promoter regions of miR-200b were amplified by genomic PCR with use of specific primers and cloned into the pGL vector directionally at Nhe*I* and Bgl*II* sites (Additional file 1: Table S1). For the promoter activity assay, empty pGL vector, pGL-miR-200b promoter, or pGL-miR-30d promoters were transfected into MPNST cells using lipofectamine 2000 (Invitrogen) reagent. Cells were then treated with vehicle only or DZNep. The pRL vector was used as an internal control. After 48 hours, cells were lysed and subjected to luciferase assays by using a dual luciferase assay kit (Promega) according to the manufacturer’s instructions.

### miRNA overexpression and reporter activity assays

To overexpress miR-30a in MPNST cells, negative control miRNA and miR-30a mimics (Dharmacon) were transfected into MPNST cells by using lipofectamine 2000. After 48 hours, cells were harvested for Western blot analyses. miR-30d and miR-200b target sequence reporters were constructed by cloning 3 repeats of miR-30d and miR-200b perfect binding sequences into the 3’ end of the luciferase gene of an empty pLightSwitch vector (SwitchGear Genomics) using Xba*I* and Xho*I* sites (Additional file 1: Table S1). The wild-type and mutant KPNB1 3’UTR reporter was generated previously [5]. For luciferase reporter analyses, luciferase reporters were transfected into MPNST cells with lipofectamine 2000. After 48 hours, reporter activity was assessed with use of LightSwitch luciferase assay reagents (SwitchGear Genomics).

### Statistical analyses

Data were analyzed by means of a two-sided unpaired *t* test using GraphPad software (Prism 6.0) and were shown as the mean ± SD of multiple independent experiments. A p value of <0.05 was considered statistically significant.

## Results

### Pharmacological inhibition of EZH2 with DZNep inhibits MPNST cell growth and induces apoptosis *in vitro*

**Figure 1:**
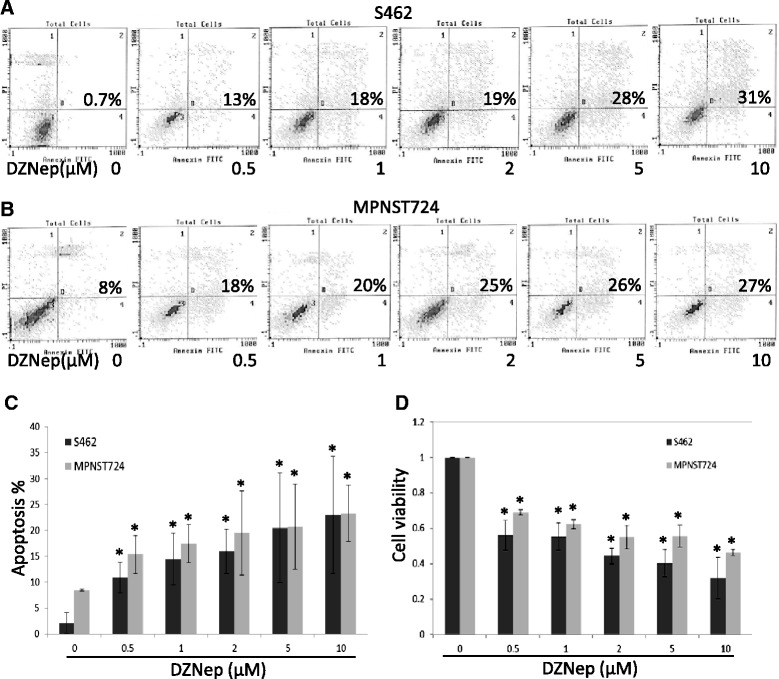
**Pharmacological inhibition of EZH2 by DZNep induced MPNST cell apoptosis. (A** and **B)** Annexin V/PI flow cytometry analyses of S462 **(A)** and MPNST724 **(B)** cells treated with DZNep at various concentrations. **(C** and **D)** Apoptosis **(C)** and cell viability **(D)** assays for S462 and MPNST724 cells treated with DZNep for 72 hours. Mean ± SD values are shown (n = 3); *p < 0.05 compared with control, Student *t* test.

Because of the importance of EZH2-regulated miR-30d expression that modulates KPNB1 in MPNST cell survival and apoptosis *in vitro* and *in vivo* [5], pharmacological inhibition of EZH2 represents a promising therapeutic approach for this tumor type. Therefore, we hypothesized that EZH2 inhibitor DZNep treatment would suppress MPNST cell proliferation and induce cell death of MPNST cells *in vitro*. To test this hypothesis, we subjected S462 and MPNST724 cells to increasing concentrations of DZNep (0, 0.5, 1, 2, 5, and 10 μM) for 72 hours and measured cell apoptosis and viability. Flow cytometry analyses of S462 and MPNST724 cells stained with annexin V and PI identified two apoptotic cell populations: early apoptotic cells (annexin V+/PI-) and late apoptotic cells (annexin V+/PI+) (Figure 1A and B). Results showed that total apoptotic cells (annexin V+), including early apoptotic cells and late apoptotic cells, increased significantly, from 0.7% to 31% for S462 cells (Figure 1A) and from 8% to 27% for MPNST724 cells (Figure 1B). In response to treatment with various doses of DZNep, the apoptotic cell populations for S462 cells increased 3 to 7 times their no-treatment control size (3% to 21% on average); the apoptotic cell populations for MPNST724 cells increased 1.5 to 2.5 times their no-treatment control size (8% to 20% on average) (Figure 1C). We then determined whether DZNep also reduced cell viability, as measured by the MTT assay (Figure 1D). Results showed that DZNep significantly reduced viability of S462 cells (from 100% to 30% on average) and MPNST724 cells (from 100% to 50% on average). We also assessed whether MPNST724 and S462 cell cycle profiles were changed by DZNep treatment. Flow cytometry cell cycle analyses with PI staining showed that for S462 cells, DZNep treatment increased the percentage of G2 phase cells and decreased the percentage of S phase cells (Additional file 2: Figure S1). For MPNST724 cells, DZNep treatment increased the percentage of G1 phase cells and decreased the percentage of S phase cells (Additional file 2: Figure S2). These data demonstrated that DZNep treatment induced cell cycle arrest and apoptosis, thereby blocking cell proliferation and diminishing survival in MPNST cells *in vitro*.

**Figure 2:**
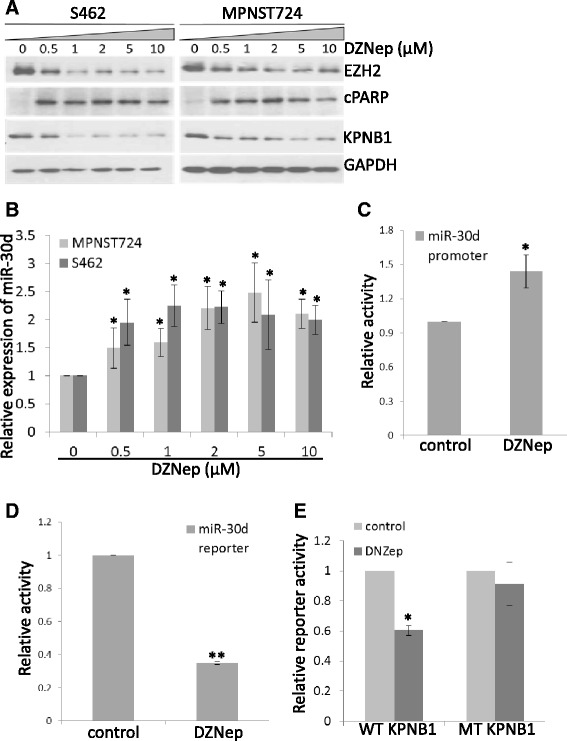
**DZNep inhibited EZH2/miR-30d/KPNB1 signaling in MPNST cells. (A)** Western blot analyses of EZH2, cleaved PARP (cPARP), KPNB1, and GAPDH (loading control) in MPNST724 and S462 cells treated with DZNep at increasing concentrations for 48 hours. **(B)** qRT-PCR analyses of miR-30d expression in MPNST724 and S462 cells treated with DZNep for 96 hours. miR-30d expression was normalized to SNORD47. Mean ± SD values are shown (n = 3); *p < 0.05, Student *t* test. **(C)** Promoter activity assay showed that DZNep treatment increased miR-30d promoter activity in S462 cells. Mean ± SD values are shown (n = 3); *p < 0.05; Student *t* test. **(D)** Luciferase reporter assay showed that DZNep treatment inhibited miR-30d target reporter activity in S462 cells. Mean ± SD values are shown (n = 3); **p < 0.01, Student *t* test. **(E)** Luciferase reporter assay indicated that DZNep treatment inhibited wild-type (WT) KPNB1 3’UTR reporter activity but not mutant (MT) KPNB1 3’UTR reporter activity in S462 cells. Mean ± SD values are shown (n = 3); *p < 0.05, Student *t* test.

On the basis of our previous study showing that EZH2 inhibits miR-30d and that miR-30d suppresses KPNB1 [5], we postulated that the EZH2 inhibitor DZNep would restore miR-30d expression with subsequent inhibition of KPNB1 expression. Under the same experimental conditions, immunoblotting revealed that EZH2 and KPNB1 expression decreased in S462 and MPNST724 cells that were treated with DZNep for 72 hours (Figure 2A). Not surprisingly, DZNep treatment increased the apoptosis marker PARP cleavage (Figure 2A). These data showed that DZNep inhibited EZH2/KPNB1 signaling in MPNST cells *in vitro*.

To determine whether EZH2 depletion by DZNep treatment also affects miR-30d expression in MPNST cells, we performed qRT-PCR analyses. Results showed that DZNep treatment restored the expression of miR-30d (Figure 2B). In our previous study, we showed that EZH2 inhibited miR-30d promoter activity in MPNST cells [5]. Here, we tested the effects of DZNep on miR-30d promoter activity in MPNST cells. We found that DZNep treatment also increased the promoter activities of miR-30d in S462 cells (Figure 2C). More importantly, we used the standard miR-30d targeting reporter construct to determine whether DZNep treatment induced functional miR-30d expression in MPNST cells. Data showed that DZNep treatment significantly inhibited miR-30d reporter activity in S462 cells (Figure 2D). Therefore, we demonstrated that DZNep induced MPNST cells apoptosis by depleting EZH2 protein and enhancing the expression and activity of miR-30d in MPNST cells.

To further confirm that DZNep-dependent KNPB1 inhibition occurs through miR-30d activation, we transfected wild-type or mutant KPNB1 3’UTR reporter constructs into S462 cells and then treated the cells with either vehicle control or DZNep. Results showed that DZNep significantly reduced the activity of the wild-type KPNB1 3’UTR reporter construct but did not affect activity levels of the mutant KPNB1 3’UTR reporter (Figure 2E). Together, these data demonstrated that DZNep depletes EZH2 expression, resulting in increased miR-30d expression and activity, which in turn inhibits KPNB1 expression in MPNST cells. These findings underscore the importance of this signaling axis in MPNST cell growth and survival.

### DZNep inhibits MPNST tumorigenesis and growth *in vivo*

**Figure 3:**
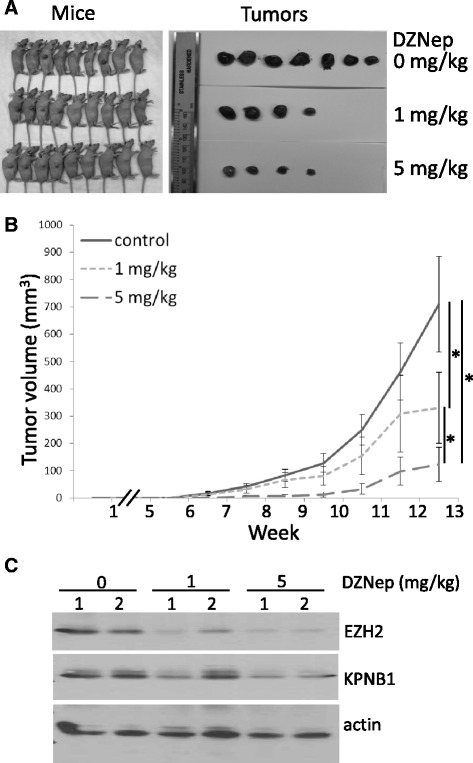
**DZNep suppressed MPNST724 xenograft tumor initiation and growth** ***in vivo*** **. (A)** MPNST724 xenograft tumor groups treated with vehicle only, 1 mg/kg DZNep, or 5 mg/kg DZNep 13 weeks after subcutaneous injection (n = 9 mice per group). **(B)** Growth curves of tumors treated with vehicle only, 1 mg/kg DZNep, or 5 mg/kg DZNep over a period of 13 weeks. Mean ± SD values are shown; *p < 0.05, Student *t* test. **(C)** Western blot analyses of EZH2, KPNB1, and actin in tumor MPNST samples treated with vehicle only, 1 mg/kg DZNep, or 5 mg/kg DZNep.

To evaluate the effects of pharmacological inhibition of EZH2 *in vivo*, we next tested whether DZNep treatment blocked MPNST tumor initiation and growth in an MPNST724 xenograft mouse model. After the subcutaneous implantation of 2 × 10^6^ MPNST724 cells, mice were treated with vehicle control only, 1 mg/kg DZNep, or 5 mg/kg DZNep intraperitoneally twice per week every other week. The doses of DZNep were chosen based on previous studies that had determined doses that are safe for mice and effective for EZH2 inhibition *in vivo* [10,13]. DZNep treatment started at the third week after cell implantation and continued for 6 weeks. The whole animal study was stopped at 13 weeks after tumor cell inoculation. Results showed that MPNST tumor initiation and growth rates were markedly suppressed by DZNep treatment. At 1 mg/kg and 5 mg/kg, DZNep decreased tumor incidence from 78% (vehicle-control group) to 44% (Figure 3A and B). The tumor volumes of the 1 mg/kg DZNep group (300 mm^3^) also significantly differed from those of the control group (700 mm^3^) (Figure 3B). In addition, tumor volumes in the 5 mg/kg group (100 mm^3^) were significantly reduced compared with those of the 1 mg/kg group (300 mm^3^) (Figure 3B).

To confirm the depletion of EZH2 in MPNST xenograft tumors by DZNep treatment *in vivo*, we conducted Western blot analyses of EZH2 and KPNB1 protein expression in two xenograft tumor samples randomly selected from each group. Data showed that DZNep decreased the EZH2 and KPNB1 protein levels of tumor samples (Figure 3C). Together, these results suggested that DZNep inhibited the EZH2/KPNB1 signaling pathway and MPNST xenograft tumor growth in mice.

**Figure 4:**
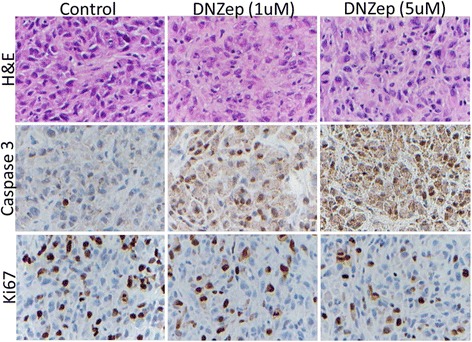
**Immunohistochemical analyses of MPNST tumors treated with control or DZNep regeant.** Represented images of H&E staining and immunohistochemical analyses of cell apoptosis marker cleaved caspase 3 and cell proliferation marker Ki-67 in MPNST724 xenograft tumor samples treated with vehicle only, 1 mg/kg DZNep, or 5 mg/kg DZNep. (×200 magnification).

Next, to determine the effects of DZNep on MPNST cell proliferation and apoptosis *in vivo*, immunohistochemical analyses of cleaved caspase 3 (apoptosis marker) and Ki-67 (cell proliferation marker) were performed. Results showed that DZNep treatment increased cleaved caspase 3 signals and decreased Ki-67 signals of xenograft tumors in a dose-dependent manner (Figure 4), suggesting that DZNep treatment induced MPNST cell apoptosis and inhibited MPNST cell proliferation *in vivo*.

### EZH2-regulated miR-30a targets KPNB1 in MPNST cells

**Figure 5:**
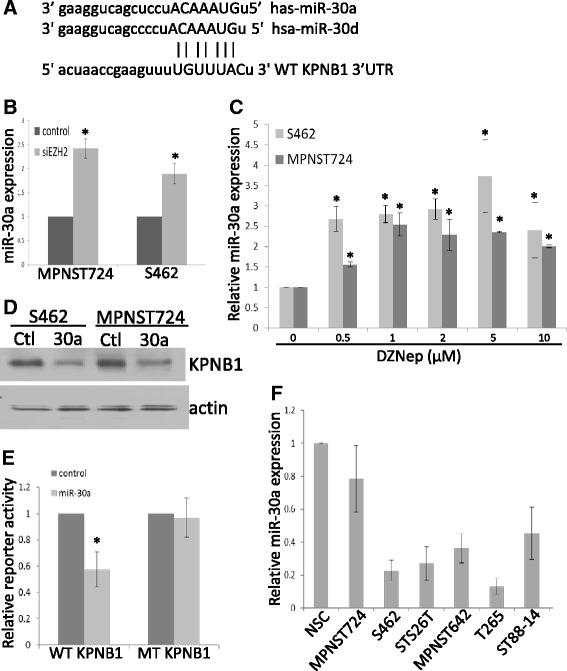
**EZH2-regulated miR-30a targeted KPNB1 in MPNST cells. (A)** Putative miR-30a and miR-30d target site in the wild-type KPNB1 3’UTR region. **(B)** qRT-PCR analyses of miR-30a in MPNST724 and S462 cells transfected with a negative control or EZH2 siRNA. miR-30a expression was normalized to SNORD47. Data are shown as mean ± SD (n = 3); *p < 0.05, Student *t* test. **(C)** qRT-PCR analyses of miR-30a in S462 and MPNST724 cells treated with DZNep for 96 hours. miR-30d expression was normalized to SNORD47. Mean ± SD values are shown (n = 3); *p < 0.05, Student *t* test. **(D)** Western blot analyses of KPNB1 and actin in S462 and MPNST724 cells transfected with a negative control (Ctl) or miR-30a (30a) mimics. **(E)** Luciferase reporter assay showed that miR-30a inhibited wild-type (WT) KPNB1 3’UTR reporter activity but not mutant (MT) KPNB1 3’UTR reporter activity in S462 cells. Mean ± SD values are shown (n = 3); *p < 0.05, Student *t* test. **(F)** qRT-PCR analyses of miR-30a expression in normal Schwann cells (NSC) and multiple MPNST cell lines (MPNST724, S462, STS26T, MPNST624, T265, and ST88-14).

In our previous study, we performed miRNA microarray analyses to identify miRNAs differentially expressed in EZH2-knockdown MPNST cells [5]. These microarray studies revealed that, in addition to miR-30d, another miR-30 family member, miR-30a, was also upregulated in EZH2-knockdown cells compared with negative controls in MPNST724, S462, and STS26T cells [5]. Because miR-30a shares the same “seed sequence” with other miR-30 family miRNAs for targeting mRNA (Figure 5A), we hypothesized that EZH2 may also regulate miR-30a and that miR-30a may inhibit KPNB1 in MPNST cells. To confirm our finding in a microarray, we conducted qRT-PCR analyses for miR-30a in an independent EZH2-knockdown experiment in MPNST724 and S462 cells. Our data showed that the miR-30a level significantly increased in EZH2-knockdown cells compared with the level in control cells (Figure 5B). Quantitative RT-PCR analyses also showed that miR-30a expression was significantly enhanced in DZNep-treated S462 and MPNST724 cells (Figure 5C).

To confirm whether miR-30a inhibits KPNB1 in MPNST cells, we transfected miR-30a mimics into MPNST724 and S462 cells. Western blot analyses indicated that the KPNB1 protein level was decreased by miR-30a mimics in both cell lines (Figure 5D). To determine whether miR-30a directly targets KPNB1 mRNA, we used a KPNB1 3’UTR reporter construct. A reporter activity assay showed that the miR-30a mimic specifically inhibited activity of the wild-type KPNB1 3’UTR reporter but not activity of a KPNB1 3’UTR reporter in which the miR-30a targeting sequence was mutated (Figure 5E). This result suggested that miR-30a directly suppresses KPNB1 by targeting the KPNB1 mRNA 3’UTR region.

To determine the expression of miR-30a in other MPNST cell lines, we performed qRT-PCR of miR-30a in a larger panel of MPNST cell lines (MPNST724, S462, STS26T, MPNST624, T265, and ST14). Results showed that miR-30a was decreased in MPNST cells compared with levels in normal Schwann cells (Figure 5 F). The expression of miR-30a had an inverse correlation with EZH2 protein and KPNB1 protein in multiple human normal Schwann cells and in the same panel of MPNST cell lines (5). These results suggested that EZH2/miR-30a/KPNB1 signaling may be common in MPNST cells.

### EZH2 regulates miR-200b expression in MPNST cells

**Figure 6:**
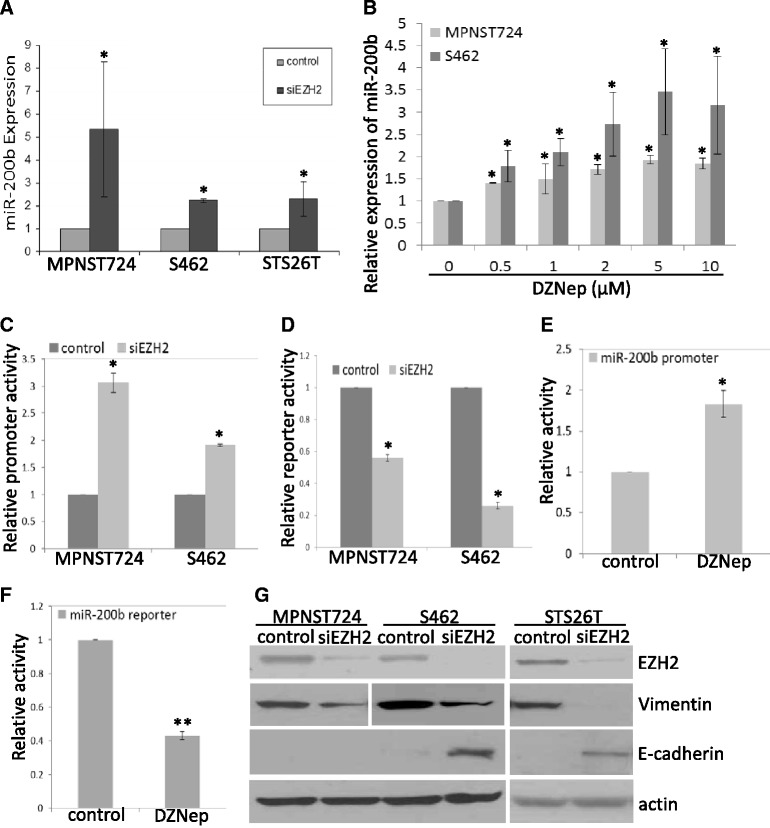
**EZH2 regulated miR-200b expression and mesenchymal-epithelial transition in MPNST cells. (A)** qRT-PCR analyses of miR-200b in MPNST724, S462, and STS26T cells transfected with a negative control or EZH2 siRNA. miR-200b expression was normalized to SNORD47. Data are shown as mean ± SD (n = 3); *p < 0.05, Student *t* test. **(B)** qRT-PCR analyses of miR-200b in S462 and MPNST724 cells treated with DZNep for 72 hours. miR-200b expression was normalized to SNORD47. Mean ± SD values are shown (n = 3); *p < 0.05, Student *t* test. **(C)** Promoter activity assay showed that EZH2 knockdown increased miR-200b promoter activity in S462 cells. Mean ± SD values are shown (n = 3); *p < 0.05, Student *t* test. **(D)** Luciferase reporter assay showed that EZH2 knockdown inhibited miR-200b target reporter activity in S462 cells. Mean ± SD values are shown (n = 3); *p < 0.05, Student *t* test. **(E)** Promoter activity assay showed that DZNep treatment induced miR-200b promoter activity in S462 cells. Mean ± SD values are shown (n = 3); *p < 0.05; Student *t* test. **(F)** Luciferase reporter assay showed that DZNep treatment suppressed miR-200b target reporter activity in S462 cells. Mean ± SD values are shown (n = 3); **p < 0.01, Student *t* test. **(G)** Western blot analyses of EZH2, vimentin, E-cadherin, and actin in MPNST724, S462, and STS26T cells transfected with a negative control or EZH2 siRNA.

Our previous miRNA microarray analyses also showed that miR-200b was upregulated when EZH2 was knocked down in MPNST724, S462, and STS26T cells [5]. We here confirmed the miR-200b expression results by qRT-PCR analyses. In an independent experiment, miR-200b expression increased significantly in EZH2-knockdown cells compared with non-targeting siRNA control cells in the MPNST724, S462, and STS26T cell lines (Figure 6A). We also determined whether the miR-200b level is increased as EZH2 is inhibited by DZNep treatment. Quantitative RT-PCR analyses showed that miR-200b expression was significantly increased by DZNep treatment in a dose-dependent manner in MPNST724 and S462 cells (Figure 6B).

To investigate the mechanism of EZH2 regulation of miR-200b, the promoter region of miR-200b was amplified from genomic DNA of MPNST cells by PCR and cloned into a pGL vector [14]. To determine whether EZH2 could inhibit miR-200b expression at the promoter level, we co-transfected non-targeting control siRNA or EZH2 siRNA with miR-200b promoter constructs into MPNST724 and S462 cells. The pRL construct was used as an internal control. Promoter activity was measured at 48 hours after transfection. Results showed that EZH2 knockdown significantly increased miR-200b promoter activity in both cell lines, suggesting that EZH2 suppresses miR-200b expression at the transcription level (Figure 6C).

We further investigated whether EZH2 knockdown induced functional miR-200b expression through the use of miR-200b target reporter systems. Based on miR-200b sequences, three repeats of miR-200b perfect match target sequences were designed and cloned into the pLightSwitch reporter vector. Therefore, these reporter constructs served as functional targets for miR-200b in transfected cells. We co-transfected control or EZH2 siRNA with miR-200b target reporter into MPNST724 and S462 cells. Luciferase activity assays showed that by silencing EZH2, miR-200b target reporter activity was significantly reduced (Figure 6D). This suggested that EZH2 knockdown induced functional miR-200b in MPNST cells.

In addition to EZH2 knockdown, we also tested the effects of EZH2 inhibitor DZNep treatment on miR-200b promoter activity and miR-200b target reporter activity in MPNST cells. Results showed that DZNep treatment significantly induced miR-200b promoter activity in S462 cells (Figure 6E). However, miR-200b target reporter activity was significantly inhibited by DZNep treatment in S462 cells (Figure 6 F). These results indicated that EZH2 suppressed miR-200b transcription in MPNST cells.

It has been shown that miR-200b reduced epithelial–mesenchymal transition by modulating vimentin and E-cadherin expression [15,16]. Therefore, we determined whether miR-200b upregulation by EZH2 siRNA had similar effects in MPNST cells. Western blot analyses showed that transient silencing of EZH2 resulted in increased E-cadherin and decreased vimentin expression in MPNST724, S462, and STS26T cells (Figure 6G). These results suggested that EZH2 knockdown may cause mesenchymal–epithelial transition in MPNST cells.

## Discussion

In this study, we found that pharmacological inhibition of EZH2 by DZNep depleted EZH2 expression, induced expression of miR-30a and miR-30d, and inhibited KPNB1 expression in MPNST cells. By impairing the EZH2/miR-30a,d/KPNB1 pathway, DNZep induced MPNST cell apoptosis and cell cycle arrest, which together decreased MPNST cell viability and suppressed cell survival *in vitro*. We also observed a consistent correlation between EZH2, miR-30a/d, and KPNB levels and MPNST cell phenotypes (apoptosis, cell cycle arrest, viability *in vitro* and tumorigenicity *in vivo*) at various concentrations of DZNep (from 0 to 10 μM). These results suggested that these phenotypes of MPNST cells induced by DZNep were mediated by EZH2/miR-30a,d/KPNB1 inhibition.

We also demonstrated that DZNep treatment inhibited MPNST tumor initiation and growth rates in a mouse xenograft model. The DZNep doses that we selected have been proven to be effective in EZH2 inhibition and safe for animals [10,13]. At high concentration of DZNep (5 mg/kg), MPNST tumors shrunk more profoundly than did tumors treated with low-dose DZNep (1 mg/kg). However, the tumors of both treatment groups did not disappear completely. These findings suggested that a higher DZNep dose may be needed for optional antitumor effects in MPNST. However, the high cost of DZNep limited our ability to perform further experiments. Nevertheless, our results suggested that EZH2 inhibition by DZNep may be a therapeutic option for MPNST.

DZNep has been identified as an inhibitor of S-adenosyl-L homocysteine hydrolase, which is required for EZH2-dependent methylation [3]. It has been shown that DZNep depletes cellular EZH2 levels and selectively blocks the trimethylation of H3K27 [9,16]. DZNep demonstrates antitumor activities against breast, lung, brain, prostate, and liver cancer cells *in vitro* [3]. More importantly, the *in vivo* efficacy of DZNep has been reported in a murine leukemia model and in a hepatocellular cancer model [10,13]. In addition, DZNep has blocked cancer cell migration and invasion in prostate cancer cells and has reduced tumor-associated blood vessel formation in a glioblastoma xenograft model [17]. However, DZNep is not considered a specific EZH2 inhibitor because it has a general histone methyltransferase inhibitory effect [7]. Several recently developed novel EZH2 small-molecule inhibitors (GSK126, EPZ-6438, and EI1) have shown potent, highly selective, and S-adenosyl-L homocysteine hydrolase competitive inhibition of wild-type and mutant EZH2 methyltransferase activity, decrease global H3K27me3 levels, and reactivate silenced PRC2 target genes [18-20]. These compounds have been shown to effectively inhibit the proliferation of multiple tumor cell lines, induce apoptosis *in vitro*, and markedly inhibit the growth of diffuse large B-cell lymphoma and pediatric malignant rhabdoid tumor xenografts in mice [18-20]. Our future studies include examining whether new EZH2 inhibitors will also have anti-MPNST effects *in vitro* and *in vivo* by using the MPNST724 mouse xenograft model.

EZH2 has been shown to be a critical regulator of epithelial-mesenchymal transition, which is a critical step for initiation of cancer invasion and metastasis [21]. EZH2 promotes EMT directly by inhibiting the expression of E-cadherin and indirectly through the NF-κB/Twist pathway [22,23]. Additionally, miR-200b is known to act as an inhibitor of EMT by targeting the transcription faction ZEB1/2 and then by activating E-cadherin [15,16]. Our study has shown that EZH2 directly inhibits miR-200b expression in MPNST, which may ultimately also contribute to EMT progression, MPNST invasion and metastasis. Interestingly, miR-30a/d have been shown to inhibit EMT and promote mesenchymal-epithelial transition of human pancreatic islet cells [24]. The miR-200 and miR-30 families have been shown to induce mesenchymal-epithelial transition [25]. Another report showed that miR-30d targets EZH2 directly [26], indicating that EZH2 and miR-30d inhibit each other and form a negative-feedback loop. Together with our findings, these data suggest that EZH2-regulated miR-200 and miR-30 family members may modulate cell survival and EMT in numerous different cancers.

## Additional files

Additional file 1: Table S1. Primers used in this study. Additional file 2
**Figure S1.** Cell cycle analyses of S462 cells treated with DZNep. **Figure S2.** Cell cycle analyses of MPNST724 cells treated with DZNep.
