# Supplementary material for: Antitumor effects of pharmacological EZH2 inhibition on malignant peripheral nerve sheath tumor through the miR-30a and KPNB1 pathway
Source: Mol Cancer. 2015 Mar 7;14:55. doi: 10.1186/s12943-015-0325-1 (PMC4357176; doi:10.1186/s12943-015-0325-1)
Supplement: Additional file 1: Table S1. — Primers used in this study. [file 12943_2015_325_MOESM1_ESM.docx]

**Additional Table 1.** Primers used in this study.

| **Primer Name** | Sequence (5'-3') |
| --- | --- |
| 3xmiR-200 target F (XbaI) | CTAGATCATCATTACCAGGCAGTATTATCATCATTACCAGGCAGTATTATCATCATTACCAGGCAGTATTAC |
| 3xmiR-200 target R (XhoI) | TCGAGTAATACTGCCTGGTAATGATGATAATACTGCCTGGTAATGATGATAATACTGCCTGGTAATGATGAT |
| 3xmiR-30d target F (XbaI) | CTAGACTTCCAGTCGGGGATGTTTACACTTCCAGTCGGGGATGTTTACACTTCCAGTCGGGGATGTTTACAC |
| 3xmiR-30d target R (XhoI) | TCGAGTGTAAACATCCCCGACTGGAAGTGTAAACATCCCCGACTGGAAGTGTAAACATCCCCGACTGGAAGT |
| miR-200 promoter -1574 F (NhEI) | ACCTGCTAGCCATTCCCGGACAGTCACAG |
| miR-200 promoter +120 R (BglII) | CAGGAGATCTCCTGGCACAGGAAGTCAGTT |
