# Supplementary material for: Antitumor effects of pharmacological EZH2 inhibition on malignant peripheral nerve sheath tumor through the miR-30a and KPNB1 pathway
Source: Mol Cancer. 2015 Mar 7;14:55. doi: 10.1186/s12943-015-0325-1 (PMC4357176; doi:10.1186/s12943-015-0325-1)
Supplement: Additional file 2 — Figure S1. Cell cycle analyses of S462 cells treated with DZNep. Figure S2. Cell cycle analyses of MPNST724 cells treated with DZNep. [file 12943_2015_325_MOESM2_ESM.pptx]

## Slide 1
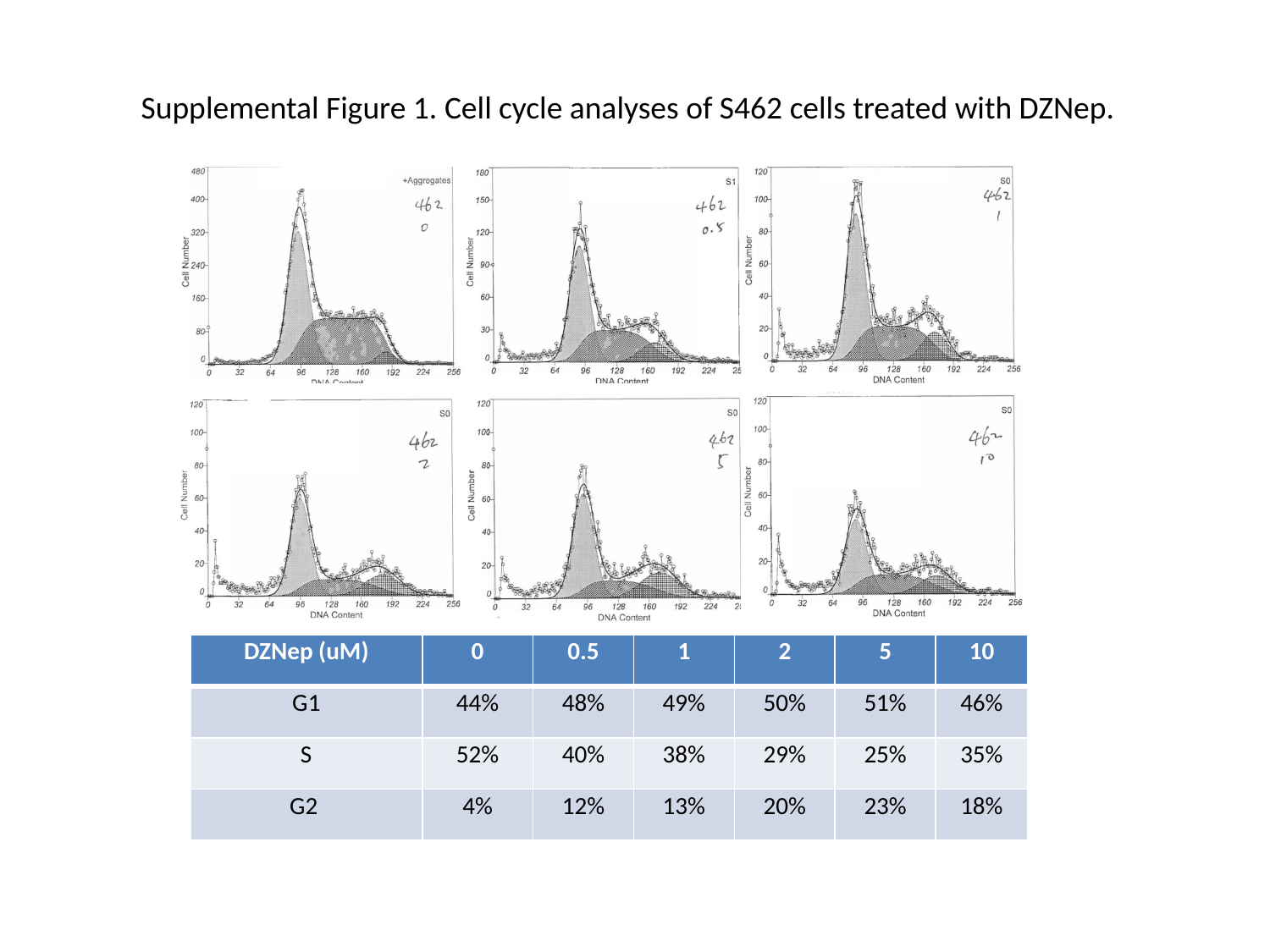

Supplemental Figure 1. Cell cycle analyses of S462 cells treated with DZNep.
| DZNep (uM) | 0 | 0.5 | 1 | 2 | 5 | 10 |
| --- | --- | --- | --- | --- | --- | --- |
| G1 | 44% | 48% | 49% | 50% | 51% | 46% |
| S | 52% | 40% | 38% | 29% | 25% | 35% |
| G2 | 4% | 12% | 13% | 20% | 23% | 18% |

## Slide 2
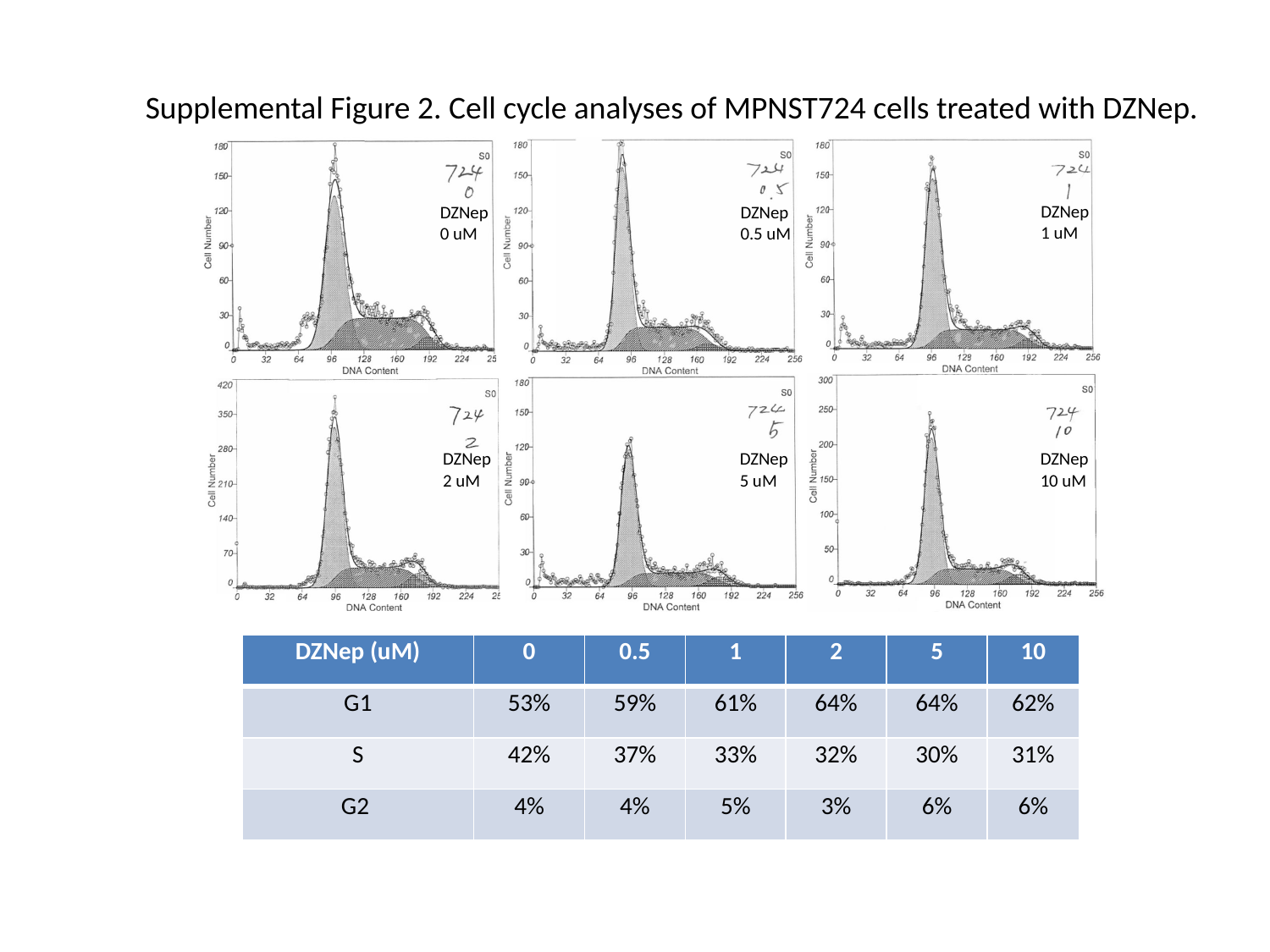

Supplemental Figure 2. Cell cycle analyses of MPNST724 cells treated with DZNep.
DZNep
1 uM
DZNep
0 uM
DZNep
0.5 uM
DZNep
2 uM
DZNep
5 uM
DZNep
10 uM
| DZNep (uM) | 0 | 0.5 | 1 | 2 | 5 | 10 |
| --- | --- | --- | --- | --- | --- | --- |
| G1 | 53% | 59% | 61% | 64% | 64% | 62% |
| S | 42% | 37% | 33% | 32% | 30% | 31% |
| G2 | 4% | 4% | 5% | 3% | 6% | 6% |
